# Crystal structure of ammonium divanadium(IV,V) tellurium(IV) hepta­oxide

**DOI:** 10.1107/S1600536814011015

**Published:** 2014-06-23

**Authors:** William T. A. Harrison, Magnus G. Johnston

**Affiliations:** a Department of Chemistry, University of Aberdeen, Meston Walk, Aberdeen AB24 3UE, Scotland

**Keywords:** crystal structure, mixed-valence, tellurium, lone-pair, layered structure

## Abstract

The polyhedral building blocks of the layered inorganic network in the mixed-valence title compound, (NH_4_)(V^IV^O_2_)(V^V^O_2_)(TeO_3_), are vertex-sharing V^V^O_4_ tetra­hedra, distorted V^IV^O_6_ octa­hedra and TeO_3_ pyramids, which are linked by V—O—V and V—O—Te bonds, forming double layers lying parallel to (100). The presumed Te^IV^ lone-pairs of electrons appear to be directed inwards into cavities in the double layers. The charge-balancing ammonium cations lie between the layers and probably inter­act with them *via* N—H⋯O hydrogen bonds.

## Abstract 2

In the title layered, mixed-valence ammonium vanadium tellurite, the V^V^ atom are tetrahedrally coordinated and the V^IV^ atoms adopt distorted octahedral coordination geometries. The presumed Te^IV^ lone pairs of electrons are directed inwards into lacunae in the double polyhedral layers.

## Chemical context   

An important feature of the crystal chemistry of tellur­ium(IV), electron configuration [Kr]4*d* ^10^5*s* ^2^, is the stereochemical activity of the 5*s* ^2^ lone-pair of electrons presumed to reside on the Te atom (Wells, 1962 ▶). This leads to distorted and unpredictable coordination polyhedra for the Te^IV^ atom in the solid state (Zemann, 1968 ▶; Weber & Schleid, 2000 ▶), and its inherent asymmetry may promote the formation of non-centrosymmetric crystal structures with potentially inter­esting physical properties (Nguyen *et al.*, 2011 ▶). As part of our studies in this area (Johnston & Harrison, 2007 ▶), we now describe the synthesis and structure of the title mixed-valence compound, (NH_4_)(V^IV^O_2_)(V^V^O_2_)(TeO_3_), (I). Some of the starting vanadium(V) was unexpectedly reduced, perhaps accompanied by oxidation of some of the ammonia.

## Structural commentary   

**Figure 1:**
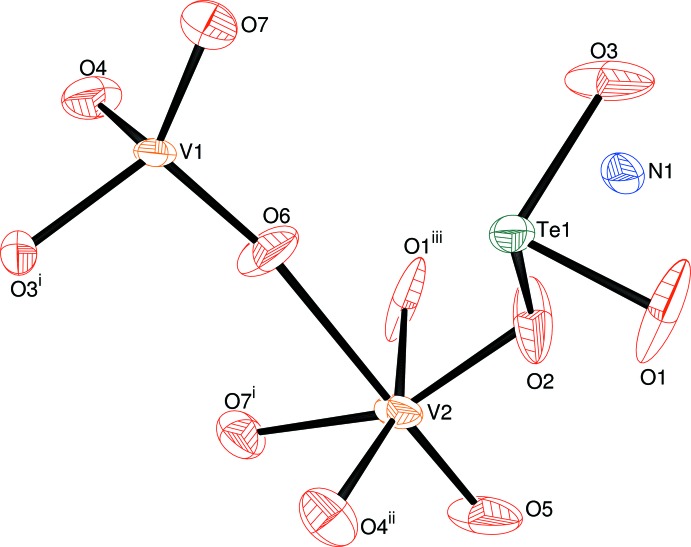
The asymmetric unit of (I) (50% displacement ellipsoids) expanded to show the coordination polyhedra of the V and Te atoms; see Table 1 ▶ for symmetry codes.

The polyhedral building units of (I) are shown in Fig. 1 ▶. Atom V1 is bonded to four O-atom neighbours (O3^i^, O4, O6 and O7; mean = 1.711 Å) in a distorted tetra­hedral arrangement (see Table 1 ▶ for symmetry codes) The mean O—V1—O bond angle is 109.2°, although the O7—V1—O3^i^ [124.1 (7)°] and O3^i^—V1—O4 [97.0 (7)°] bond angles diverge considerably from the ideal tetra­hedral value. The bond-valence-sum (BVS) values (in valence units) for V1, as calculated by the Brown & Altermatt (1985 ▶) formalism, using parameters appropriate for V^IV^ and V^V^, are 4.96 and 5.22, respectively. Both clearly indicate a penta­valent state for this atom.

The coordination polyhedron about atom V2 is a distorted octa­hedron. O5 is bonded to V2 by a short ‘vanad­yl’ V=O double bond [1.612 (5) Å], whilst O1, O4, O7 and O2 occupy the equatorial positions with V—O bond lengths between 1.93 and 2.06 Å. O6 is located *trans* to O5 [O5—V2—O6 = 176.1 (11)°] and is consequently much farther away from the metal ion [2.311 (5) Å] than the other O atoms. This octa­hedral distortion mode is characteristic of both vanadium(IV) and vanadium(V) and may be theoretically analysed in terms of a second-order Jahn–Teller distortion (Kunz & Brown, 1995 ▶). The O—V2—O bond angles also show a broad spread [*cis*: 73.8 (5) to 104.2 (8)°, *trans*: 157.0 (6) to 176.1 (11)°]. BVS calculations for V2 yield values of 4.20 (V^IV^ parameters) and 4.42 (V^V^ parameters), which both indicate vanadium(IV).

Te1 is three-coordinated by oxygen atoms (O1, O2 and O3) in a distorted trigonal–pyramidal arrangement [mean Te–O = 1.867 Å; BVS(Te1) = 3.98]. The O—Te—O bond angles are all less than 95°, suggesting that a treatment of the bonding about Te involving *sp* ^3^ hybrid orbitals and a lone pair (as in ammonia) may be too simple (Wells, 1962 ▶). As is typical (Feger *et al.*, 1999 ▶) of the crystal chemistry of tellurium(IV), its environment includes further O atoms much closer than the Bondi (1964 ▶) van der Waals radius sum of 3.65 Å for Te and O. In particular, there is a fourth O atom within 2.70 Å [Te1—O7^vii^ = 2.695 (7) Å (vii) = 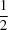 − *x*, 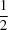 + *y*, 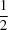 + *z*], which results in an overall distorted folded-square arrangement about Te1.

Assuming the presence of V^V^ and V^IV^ in equal amounts in the structure, the charge-balancing criterion indicates that N1 must be part of an ammonium ion (which is obviously consistent with the use of significant qu­anti­ties of ammonia in the synthesis), although no H atoms could be located from the present diffraction data. However, short N⋯O contacts in the crystal structure (*vide infra*) are indicative of hydrogen bonding. The presence of NH_4_^+^ ions is also supported by the IR spectrum of (I). The alternative possibilities of neutral ammonia mol­ecules or water mol­ecules and a different distribution of vanadium oxidation states seem far less likely to us.

## Packing features   

**Figure 2:**
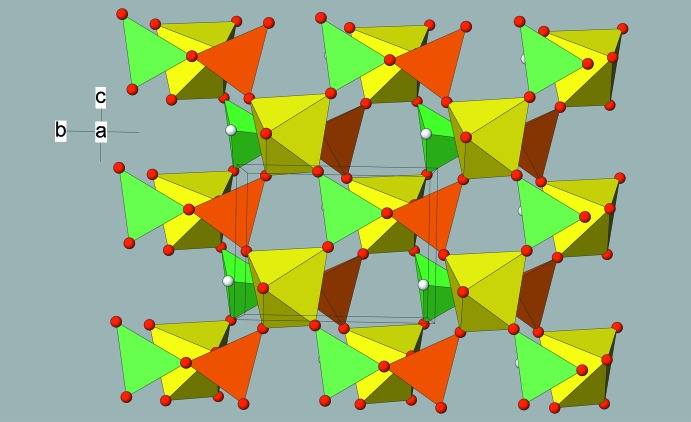
View approximately down [100] of part of a polyhedral layer in (I). Colour key: V1O_4_ tetra­hedra orange, V2O_6_ octa­hedra yellow, O atoms red. The TeO_3_ pyramids are shown as green pseudo-tetra­hedra with the presumed lone-pair of electrons shown as a white sphere.

The connectivity of the VO_4_, VO_6_ and TeO_3_ polyhedra in (I) leads to a layered structure. The building blocks share vertices *via* V—O—V and V—O—Te bonds; conversely, there are no Te—O—Te links, which can occur in tellurium-rich compounds (Irvine *et al.*, 2003 ▶). Each anionic layer in (I) is constructed from two infinite (100) sheets of composition [(V^IV^O_2_)(V^V^O_2_)(TeO_3_)]^−^, built up from a network of corner-sharing four- and six-membered rings (Fig. 2 ▶). The four-membered rings are built from one TeO_3_, one V1O_4_ tetra­hedron and two V2O_6_ octa­hedra, whilst the six-membered rings are constructed from two of each different polyhedra. It is inter­esting to note the V—O—V inter-polyhedral angles (mean = 154.1°) are much more obtuse than the Te—O—V angles (mean = 124.0°).

The two sheets within each layer are linked through V2—O6—V1 bonds and are orientated so that the four-membered rings of one sheet are aligned with the six-membered rings of the other, and the lone-pair electrons of the Te^IV^ species point into the centre of the layer. These ‘lone-pairs sandwiches’ represent a novel way of accommodating the Te^IV^ lone-pairs, which may be compared to self-contained ‘tubes’ in BaTe_3_O_7_ and BaTe_4_O_9_ (Johnston & Harrison, 2002 ▶) or large 12-ring channels in Mg_0.5_ZnFe(TeO_3_)_3_·4.5H_2_O (Miletich, 1995 ▶).

**Figure 3:**
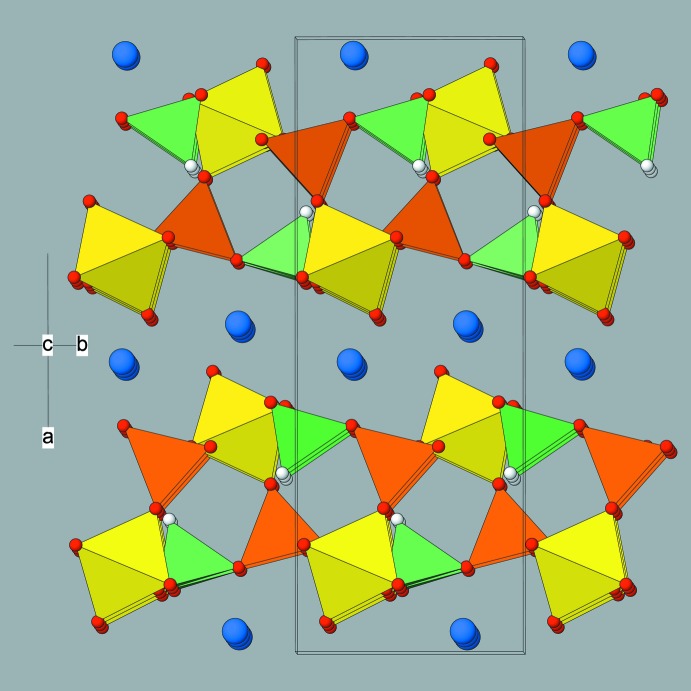
View approximately down [001] of the crystal structure of (I) showing the (100) polyhedral layers inter­spersed by ammonium ions. Colour key: N atoms blue, other atoms as in Fig. 2 ▶.

The layers stack in the [100] direction, with the ammonium cations occupying the inter-layer regions (Fig. 3 ▶). Connectivity between the layers is presumably mediated by N—H⋯O hydrogen bonds, with N1 having eight O-atom neighbours within 3.4 Å (four in each layer). The N⋯O distances are listed in Table 2 ▶.

## Database survey   

A search of the Inorganic Crystal Structure Database (ICSD, 2014 ▶; web version 2.2.2) revealed three compounds containing ammonium ions, vanadium, tellurium and oxygen: (NH_4_)_2_(VO_2_)[TeO_4_(OH)]·H_2_O (Kim *et al.*, 2007 ▶) contains V^V^O_4_ tetra­hedra and Te^VI^O_5_(OH) octa­hedra, which link together into infinite chains. (NH_4_)_2_(VO_2_)_2_[TeO_4_(OH_2_)] (Yun *et al.*, 2010 ▶) is a layered structure containing unusual V^V^O_5_ square pyramids and Te^VI^O_4_(OH_2_) octa­hedra. (NH_4_)_9_K(Mo_12_V_12_TeO_69_)(TeO_3_)_2_·27H_2_O (Corella-Ochoa *et al.*, 2011 ▶) is a complex polyoxidometallate containing V^V^, V^IV^ and Te^IV^ atoms.

## Synthesis and crystallization   

0.7276 g (4 mmol) of V_2_O_5_ and 0.3249 g (3 mmol) TeO_2_ were placed in a 23 ml capacity Teflon-lined stainless steel autoclave. Added to this were 7 ml of a 1.3 *M* NH_3_ solution and 8 ml of H_2_O (pre-oven pH = 8.5). The autoclave was sealed and heated in an oven at 438 K for three days, followed by cooling to room temperature over a few hours. The resulting solid products, consisting of dark-red needles of (I), transparent chunks of TeO_2_ and an unidentified yellow powder, were recovered by vacuum filtration and washing with water and acetone. IR data (KBr disk) were collected using a hand-picked sample of (I): broad bands at ∼3400 and 3000 cm^−1^ can be ascribed to the symmetric and asymmetric stretches of the tetra­hedral ammonium ion (Balraj & Vidyasagar, 1998 ▶). The doublet at 1440 and 1411 cm^−1^ is indicative of H—N—H bending modes; the presence of a doublet is in itself inter­esting, suggesting there may be some disorder associated with the H atoms of the ammonium cation. This phenomenon may also contribute to the difficulty in locating the H-atom positions from the X-ray data. The large number of overlapping bands in the 1000–400 cm^−1^ range can be attributed to framework V=O, V—O, Se—O and O—Se—O modes.

## Refinement   

Crystal data, data collection and structure refinement details are summarized in Table 3 ▶. The H atoms could not be located in difference maps, neither could they be geometrically placed. The crystal studied was found to be a racemic twin.

## Supplementary Material

Crystal structure: contains datablock(s) I. DOI: 10.1107/S1600536814011015/wm0003sup1.cif

Structure factors: contains datablock(s) I. DOI: 10.1107/S1600536814011015/wm0003Isup2.hkl

CCDC reference: 1004307

Additional supporting information:  crystallographic information; 3D view; checkCIF report

## Figures and Tables

**Table 1:** Selected geometric parameters (Å, °)

V1—O7	1.631 (7)	V2—O1^iii^	1.973 (16)
V1—O6	1.656 (5)	V2—O7^i^	2.053 (7)
V1—O3^i^	1.770 (5)	V2—O6	2.311 (5)
V1—O4	1.788 (9)	Te1—O1	1.748 (14)
V2—O5	1.612 (5)	Te1—O3	1.921 (5)
V2—O2	1.935 (15)	Te1—O2	1.931 (14)
V2—O4^ii^	1.961 (7)		
			
Te1—O1—V2^iv^	125.5 (9)	V1—O4—V2^vi^	145.3 (4)
Te1—O2—V2	120.3 (8)	V1—O6—V2	167.7 (6)
V1^v^—O3—Te1	131.3 (2)	V1—O7—V2^v^	149.9 (4)

Symmetry codes: (i) 
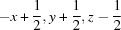
; (ii) 
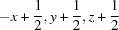
; (iii) 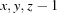; (iv) 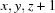; (v) 
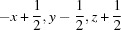
; (vi) 
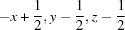
.

**Table 2:** Hydrogen-bond geometry (Å)

*D*—H⋯*A*	*D*⋯*A*	*D*—H⋯*A*	*D*⋯*A*
N1⋯O5^vii^	2.820 (7)	N1⋯O5^viii^	3.15 (3)
N1⋯O1^iii^	2.89 (2)	N1⋯O1^viii^	3.20 (2)
N1⋯O2	2.95 (2)	N1⋯O5^ix^	3.20 (3)
N1⋯O2^viii^	2.96 (2)	N1⋯O3^iii^	3.39 (3)

Symmetry codes: (iii) 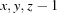; (vii) 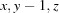; (viii) 
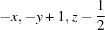
; (ix) 
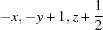
.

**Table 3:** Experimental details

Crystal data	
Chemical formula	(NH_4_)(VO_2_)(VO_2_)(TeO_3_)
*M*_r_	359.52
Crystal system, space group	Orthorhombic, *P* *n* *a*2_1_
Temperature (K)	293
*a*, *b*, *c* (Å)	18.945 (2), 7.0277 (8), 5.4402 (6)
*V* (Å^3^)	724.29 (14)
*Z*	4
Radiation type	Mo *K*α
μ (mm^−1^)	6.52
Crystal size (mm)	0.17 × 0.02 × 0.02

Data collection	
Diffractometer	Bruker *SMART1000* CCD
Absorption correction	Multi-scan (*SADABS*; Bruker, 2000 ▶)
*T*_min_, *T*_max_	0.404, 0.881
No. of measured, independent and observed [*I* > 2σ(*I*)] reflections	7528, 2368, 1595
*R*_int_	0.047
(sin θ/λ)_max_ (Å^−1^)	0.756

Refinement	
*R*[*F* ^2^ > 2σ(*F* ^2^)], *wR*(*F* ^2^), *S*	0.040, 0.082, 0.98
No. of reflections	2368
No. of parameters	101
No. of restraints	1
Δρ_max_, Δρ_min_ (e Å^−3^)	0.99, −1.13
Absolute structure	Flack (1983 ▶), 1201 Friedel pairs
Absolute structure parameter	0.5 (1)

Computer programs: *SMART* and *SAINT* (Bruker, 2000 ▶), *SHELXS97* and *SHELXL97* (Sheldrick, 2008 ▶), *ORTEP-3 for Windows* (Farrugia, 2012 ▶) and *ATOMS* (Dowty, 1999 ▶).

## supplementary crystallographic information

### Crystal data

**Table:**

(NH_4_)(VO_2_)_2_(TeO_3_)	*D*_x_ = 3.297 Mg m^−^^3^
*M**_r_* = 359.52	Mo *K*α radiation, λ = 0.71073 Å
Orthorhombic, *P**n**a*2_1_	Cell parameters from 5060 reflections
*a* = 18.945 (2) Å	θ = 2.2–32.5°
*b* = 7.0277 (8) Å	µ = 6.52 mm^−^^1^
*c* = 5.4402 (6) Å	*T* = 293 K
*V* = 724.29 (14) Å^3^	Rod, dark red
*Z* = 4	0.17 × 0.02 × 0.02 mm
*F*(000) = 660	

### Data collection

**Table:**

Bruker SMART1000 CCD diffractometer	2368 independent reflections
Radiation source: fine-focus sealed tube	1595 reflections with *I* > 2σ(*I*)
Graphite monochromator	*R*_int_ = 0.047
ω scans	θ_max_ = 32.5°, θ_min_ = 2.2°
Absorption correction: multi-scan (*SADABS*; Bruker, 2000)	*h* = −28→26
*T*_min_ = 0.404, *T*_max_ = 0.881	*k* = −10→10
7528 measured reflections	*l* = −6→8

### Refinement

**Table:**

Refinement on *F*^2^	Secondary atom site location: difference Fourier map
Least-squares matrix: full	Hydrogen site location: notdet
*R*[*F*^2^ > 2σ(*F*^2^)] = 0.040	*w* = 1/[σ^2^(*F*_o_^2^) + (0.0318*P*)^2^] where *P* = (*F*_o_^2^ + 2*F*_c_^2^)/3
*wR*(*F*^2^) = 0.082	(Δ/σ)_max_ < 0.001
*S* = 0.98	Δρ_max_ = 0.99 e Å^−^^3^
2368 reflections	Δρ_min_ = −1.13 e Å^−^^3^
101 parameters	Absolute structure: Flack (1983), 1201 Friedel pairs
1 restraint	Absolute structure parameter: 0.5 (1)
Primary atom site location: structure-invariant direct methods	

### Special details

**Table:**

Geometry. All e.s.d.'s (except the e.s.d. in the dihedral angle between two l.s. planes) are estimated using the full covariance matrix. The cell e.s.d.'s are taken into account individually in the estimation of e.s.d.'s in distances, angles and torsion angles; correlations between e.s.d.'s in cell parameters are only used when they are defined by crystal symmetry. An approximate (isotropic) treatment of cell e.s.d.'s is used for estimating e.s.d.'s involving l.s. planes.
Refinement. Refinement of *F*^2^ against ALL reflections. The weighted *R*-factor *wR* and goodness of fit *S* are based on *F*^2^, conventional *R*-factors *R* are based on *F*, with *F* set to zero for negative *F*^2^. The threshold expression of *F*^2^ > σ(*F*^2^) is used only for calculating *R*-factors(gt) *etc*. and is not relevant to the choice of reflections for refinement. *R*-factors based on *F*^2^ are statistically about twice as large as those based on *F*, and *R*- factors based on ALL data will be even larger.

### Fractional atomic coordinates and isotropic or equivalent isotropic displacement parameters (Å^2^)

**Table:**

	*x*	*y*	*z*	*U*_iso_*/*U*_eq_	
N1	0.0293 (3)	0.2583 (8)	0.225 (5)	0.031 (2)	
V1	0.31266 (5)	0.53486 (13)	0.2408 (12)	0.0245 (2)	
V2	0.12159 (5)	0.75777 (13)	0.2385 (9)	0.0238 (2)	
Te1	0.164135 (17)	0.50920 (5)	0.7434 (5)	0.02034 (10)	
O1	0.1064 (8)	0.561 (3)	0.985 (2)	0.056 (5)	
O2	0.0970 (8)	0.575 (3)	0.490 (2)	0.048 (4)	
O3	0.1367 (2)	0.2463 (7)	0.728 (4)	0.053 (2)	
O4	0.3316 (4)	0.4539 (9)	−0.0638 (13)	0.0355 (15)	
O5	0.0453 (3)	0.8594 (7)	0.231 (5)	0.055 (2)	
O6	0.2292 (2)	0.6033 (8)	0.224 (4)	0.043 (2)	
O7	0.3270 (3)	0.3621 (9)	0.4347 (13)	0.0332 (14)	

### Atomic displacement parameters (Å^2^)

**Table:**

	*U*^11^	*U*^22^	*U*^33^	*U*^12^	*U*^13^	*U*^23^
N1	0.020 (2)	0.021 (3)	0.053 (6)	−0.004 (2)	0.000 (7)	0.005 (7)
V1	0.0150 (4)	0.0096 (4)	0.0488 (7)	0.0017 (3)	−0.003 (2)	0.003 (3)
V2	0.0200 (4)	0.0128 (4)	0.0386 (6)	−0.0008 (3)	−0.0089 (16)	−0.003 (2)
Te1	0.01691 (14)	0.01447 (16)	0.02964 (19)	0.00084 (13)	0.0000 (9)	−0.0019 (7)
O1	0.017 (5)	0.099 (10)	0.052 (8)	−0.022 (5)	0.018 (4)	−0.054 (7)
O2	0.018 (5)	0.088 (9)	0.040 (8)	−0.011 (5)	−0.006 (4)	0.038 (6)
O3	0.028 (2)	0.018 (2)	0.114 (7)	0.003 (2)	−0.037 (7)	−0.004 (8)
O4	0.043 (4)	0.029 (3)	0.035 (3)	0.012 (3)	0.005 (3)	−0.002 (3)
O5	0.030 (2)	0.020 (2)	0.115 (6)	0.007 (2)	−0.025 (9)	−0.005 (11)
O6	0.018 (2)	0.040 (3)	0.070 (6)	0.0099 (19)	−0.008 (6)	−0.021 (7)
O7	0.036 (3)	0.027 (3)	0.037 (4)	0.003 (3)	0.002 (3)	0.010 (3)

### Geometric parameters (Å, º)

**Table:**

V1—O7	1.631 (7)	V2—O6	2.311 (5)
V1—O6	1.656 (5)	Te1—O1	1.748 (14)
V1—O3^i^	1.770 (5)	Te1—O3	1.921 (5)
V1—O4	1.788 (9)	Te1—O2	1.931 (14)
V2—O5	1.612 (5)	O1—V2^iv^	1.973 (16)
V2—O2	1.935 (15)	O3—V1^v^	1.770 (5)
V2—O4^ii^	1.961 (7)	O4—V2^vi^	1.961 (7)
V2—O1^iii^	1.973 (16)	O7—V2^v^	2.053 (7)
V2—O7^i^	2.053 (7)		
			
O7—V1—O6	114.3 (6)	O1^iii^—V2—O7^i^	75.9 (6)
O7—V1—O3^i^	124.1 (7)	O5—V2—O6	176.1 (11)
O6—V1—O3^i^	105.7 (3)	O2—V2—O6	85.7 (6)
O7—V1—O4	109.2 (4)	O4^ii^—V2—O6	87.1 (4)
O6—V1—O4	103.4 (8)	O1^iii^—V2—O6	77.0 (6)
O3^i^—V1—O4	97.0 (7)	O7^i^—V2—O6	73.8 (5)
O5—V2—O2	95.6 (9)	O1—Te1—O3	93.7 (9)
O5—V2—O4^ii^	96.2 (5)	O1—Te1—O2	94.2 (3)
O2—V2—O4^ii^	100.8 (6)	O3—Te1—O2	91.2 (7)
O5—V2—O1^iii^	99.2 (8)	Te1—O1—V2^iv^	125.5 (9)
O2—V2—O1^iii^	89.7 (3)	Te1—O2—V2	120.3 (8)
O4^ii^—V2—O1^iii^	160.3 (5)	V1^v^—O3—Te1	131.3 (2)
O5—V2—O7^i^	104.2 (8)	V1—O4—V2^vi^	145.3 (4)
O2—V2—O7^i^	157.0 (6)	V1—O6—V2	167.7 (6)
O4^ii^—V2—O7^i^	88.6 (3)	V1—O7—V2^v^	149.9 (4)

Symmetry codes: (i) −*x*+1/2, *y*+1/2, *z*−1/2; (ii) −*x*+1/2, *y*+1/2, *z*+1/2; (iii) *x*, *y*, *z*−1; (iv) *x*, *y*, *z*+1; (v) −*x*+1/2, *y*−1/2, *z*+1/2; (vi) −*x*+1/2, *y*−1/2, *z*−1/2.

### Hydrogen-bond geometry (Å)

**Table:**

*D*—H···*A*	*D*···*A*
N1···O5^vii^	2.820 (7)
N1···O1^iii^	2.89 (2)
N1···O2	2.95 (2)
N1···O2^viii^	2.96 (2)
N1···O5^viii^	3.15 (3)
N1···O1^viii^	3.20 (2)
N1···O5^ix^	3.20 (3)
N1···O3^iii^	3.39 (3)

Symmetry codes: (iii) *x*, *y*, *z*−1; (vii) *x*, *y*−1, *z*; (viii) −*x*, −*y*+1, *z*−1/2; (ix) −*x*, −*y*+1, *z*+1/2.
